# Translating it into real life: a qualitative study of the cognitions, barriers and supports for key obesogenic behaviors of parents of preschoolers

**DOI:** 10.1186/s12889-015-1554-3

**Published:** 2015-02-26

**Authors:** Jennifer Martin-Biggers, Kim Spaccarotella, Nobuko Hongu, Gayle Alleman, John Worobey, Carol Byrd-Bredbenner

**Affiliations:** Department of Nutritional Sciences, Rutgers, The State University of New Jersey, 26 Nichol Avenue, New Brunswick, NJ 08901 USA; Department of Biological Sciences, Kean University, 1000 Morris Avenue, Union, NJ 07082 USA; Department of Nutritional Sciences, University of Arizona, 406 Shantz Building, 1177 E. 4th Street, Tucson, AZ 85721-0038 USA

**Keywords:** Home environment, Obesity, Preschoolers

## Abstract

**Background:**

Little is known about preschool parents’ cognitions, barriers, supports and modeling of key obesogenic behaviors, including breakfast, fruit and vegetable consumption, sugary beverage intake, feeding practices, portion sizes, active playtime, reduced screen-time, sleep and selection of child-care centers with characteristics that promote healthy behaviors.

**Methods:**

Thus, the purpose of this study was to examine these factors via survey and focus groups among 139 parents of 2- to 5-year-old children. Standard content analysis procedures were used to identify trends and themes in the focus group data, and Analysis of Variance was used to test for differences between groups in the survey data.

**Results:**

Results showed 80% of parents ate breakfast daily, consumed sugary beverages 2.7 ± 2.5SD days per week, and had at least two different vegetables and fruits an average of 5.2 ± 1.8SD and 4.6 ± 2.0SD days per week. Older parents and those with greater education drank significantly fewer sugary drinks. Parents played actively a mean 4.2 ± 2.2 hours/week with their preschoolers, who watched television a mean 2.4 ± 1.7 hours/day. Many parents reported having a bedtime routine for their preschooler and choosing childcare centers that replaced screen-time with active play and nutrition education. Common barriers to choosing healthful behaviors included lack of time; neighborhood safety; limited knowledge of portion size, cooking methods, and ways to prepare healthy foods or play active indoor games; the perceived cost of healthy options, and family members who were picky eaters. Supports for performing healthful behaviors included planning ahead, introducing new foods and behaviors often and in tandem with existing preferred foods and behaviors, and learning strategies from other parents.

**Conclusions:**

Future education programs with preschool parents should emphasize supports and encourage parents to share helpful strategies with each other.

## Background

In the U.S., approximately 22.8% of preschool-aged children (i.e., 2- to 5-years-old) are overweight or obese (Body Mass Index, BMI, for age ≥85^th^ percentile), with 8.4% already obese (BMI for age ≥95^th^ percentile) [1]. Recent data indicate that the prevalence of obesity has declined or remained stable among preschool children [2,3], yet obesity remains an important public health problem. Overweight during childhood sets the stage for premature development of chronic diseases [4-7]. Obese children also experience psychological stress in the form of social stigmatization and depression [8-11].

Social Cognitive Theory posits that parent modeling of behaviors, including weight-related behaviors, is important for teaching young children positive habits [12]. Indeed, evidence indicates that, as role models and gatekeepers, parents strongly influence weight-related behaviors of children [9,13-21] and are key players in obesity prevention [22-25]. For example, the influence of parental modeling of screen-time physical activity, beliefs about physical activity, and encouragement provided are important predictors of children’s physical activity levels [13,14,26-30]. Parent food practices (e.g., child feeding strategies, breakfast consumption, sugar-sweetened beverage intake) and sleep habits also influence child behaviors [20,26,31,32]. In addition, children’s intakes of and preferences for vegetables, fruits, and calcium-rich foods are positively associated with availability at home, parental intake, and allowing the child to decide how much of the food to eat [33-42].

Because BMI, as well as behaviors that directly affect BMI (i.e., diet, physical activity, and sleep habits), track across childhood into adulthood, it is important for parents to actively safeguard children’s health by establishing positive obesity-preventive lifestyle habits [43-52]. However, little is known about parents’ modeling of key weight-related behaviors, barriers to performing these behaviors, and strategies to overcome barriers. Few studies [53-55] comprehensively investigate parents’ cognitions associated with key obesity-prevention behaviors (i.e., attitudes, barriers and strategies for overcoming barriers to breakfast, fruit and vegetable consumption, reduced sugar-sweetened beverage intake, positive feeding practices, active playtime, reduced screen-time, sleep and selection of childcare with characteristics that support healthy behaviors). Thus, the purpose of this study was to examine these factors quantitatively and qualitatively with parents of 2- to 5-year-old children.

## Methods

This study was approved by the Institutional Review Boards at Rutgers, the State University of New Jersey and the University of Arizona. All participants gave informed consent.

### Sample

Parents of 2- to 5-year-old children whose primary language was English or Spanish were recruited via flyers posted at community sites and emails sent from workplace listservs in New Jersey and Arizona. These states were chosen because at the time of planning the study, the rate of obesity among preschoolers in New Jersey was among the highest in the United States and Arizona had recently reported the largest increase in preschooler obesity prevalence of all states [56]. Recruitment notices invited parents to participate in a 60-minute discussion about small, simple changes at home that can help their children grow up healthier and offered $25 compensation.

### Instruments

Each parent completed a survey and participated in a focus group. The survey gathered data related to demographic characteristics (e.g., age, highest education level, number and ages of children) and key weight-related behaviors, including weekly frequency of family meal, fruit, vegetable, sugar-sweetened beverage and breakfast consumption, parental physical activity, preschooler screen-time, feeding strategies, bedtime routines and food insecurity (e.g., “How many days per week do you eat breakfast?”, “Do you have a set bedtime in your home for your preschool children?”).

Focus group moderators were trained researchers fluent in the primary language of the participants (i.e., English and Spanish groups were conducted separately). A structured moderator’s protocol was created using standard guidelines for conducting focus groups [57,58] and used to conduct the focus groups. Each focus group included questions on a maximum of two, randomly selected weight-related topics associated with childhood obesity: breakfast consumption, sugar-sweetened beverage intake, fruits and vegetable intake, parent feeding practices, active play behaviors, screen-time behaviors, and/or parental communications with child care providers about parents’ preferred weight-related behavioral practices. The focus group participants were asked a series of questions, based on key Social Cognitive Theory constructs, designed to elucidate their attitudes toward the weight-related topic, identify barriers to performing recommended behaviors associated with the weight-related topic, and discover strategies for overcoming barriers. For instance, focus groups addressing fruits and vegetables examined parents’ attitudes toward eating these foods, barriers to serving these foods, and strategies parents use to overcome barriers to serving these foods. A second trained moderator took comprehensive notes at each focus group. Within 48 hours of the end of each focus group, the second moderator transcribed the notes, which then were reviewed by the focus group moderator for clarity, thoroughness, and accuracy. Spanish language notes were translated into English by the researchers present at each group. Inconsistencies in translation were examined, and consensus was reached by the researchers present during the particular focus group.

### Data analysis

Survey data were analyzed using SPSS version 21.0 (Chicago, IL). Analysis of Variance (ANOVA) was used to test for differences between groups (primary language spoken, geographic location, parent education level, parent age, and food security/insecurity). Differences were considered significant at *p* < 0.05. Values are reported as means and standard deviations unless otherwise noted.

Standard content analysis procedures were used by three trained researchers to identify trends and themes in the focus group data [59,60]. Content analysis techniques generate objective, systematic, and quantitative descriptions [61,62] from which researchers can draw “replicable and valid inferences from the data to their context”[63], p. 21. Researchers compared their independent analyses and discussed differences to reach a unanimous agreement. The constant data comparison method was utilized concurrently with data collection to ascertain when data saturation (or information redundancy) for each weight-related topic was realized and data collection should terminate [60,64].

## Results

A total of 139 parents completed the survey and participated in 1 of 43 focus group interviews (mean focus group size = 3.23 persons). Participants had a mean of 2.29 ± 1.15SD children, and the mean age of parents was 32.18 ± 7.12SD years. About two-thirds had received at least some college education (n = 47 high school or less; n = 48 some post-secondary; n = 42 baccalaureate or higher; n = 2 no response) and most (60%) spoke English whereas 40% spoke Spanish. Thirty-seven of the participants reported food insecurity during the past year. Participants were approximately evenly distributed between geographic locations (n = 73 NJ, n = 66 AZ), and all topics were discussed by focus groups in both states.

### Breakfast consumption

Survey results indicated 80% of parents (n = 138) ate breakfast daily, whereas 8% ate breakfast one day or less weekly. Breakfast eating frequency did not differ by primary language spoken, geographic location, education level, age, or food security.

### Attitudes toward breakfast consumption

Parents in the 9 breakfast focus groups (n = 23) felt breakfast was important because it supplies energy (“*Breakfast is where our energy for the day starts so it*’*s important to eat breakfast to get ready for the day*”) and nutrients (“*nutritional factor and helpful for growth*”), affects overall eating patterns (“*it sets the eating schedule for the rest of the day*”), and has satiety value (feel “*more full throughout the day*”). Parents also felt breakfast was important because children need it for “*focusing in school*” and to be “*ready to roll and see their friends*”. Parents saw breakfast as a time for the family to be together (“*We try to make breakfast important since it is time to spend together*”) and a way to manage child behavior (“*If my kid doesn*’*t eat*, *he*’*ll get grumpy*”).

They described a good breakfast as including whole grains and fruit; some were uncertain about whether ready-to-eat cereal was a healthy option. During the week, parents relied on quick-to-prepare foods, but healthfulness of foods served varied (“*Coffee and a pastry*, *but I know that*’*s a bad habit*”). On weekends, parents reported they had the opportunity to eat foods needing more time to prepare, such as eggs, bacon, and pancakes.

### Perceived barriers to breakfast consumption

The most common barrier to having breakfast was morning time stress, which makes it “*hard to make breakfast a priority*”. Work schedules and household chaos (“*We are running around the house before school*”) were commonly named contributors to time stress. Another important barrier was lack of planning or forgetfulness (“*forgets to buy milk*”, “*not having food at home*”) or not having foods on hand that could be prepared and served quickly.

### Strategies for overcoming barriers to breakfast consumption

When asked what helps get kids to eat breakfast, parents reported using several strategies, including time management techniques, like waking earlier, preparing the night before (e.g., putting bowls and cereal boxes on the counter; being sure “*clothes are out for tomorrow*”, “*shower at night*”; “*dice up fruit and put it in the fridge so it is ready for the kids to eat*”) and dovetailing (“*pack a small breakfast with lunch*”). Parents who overcame barriers to breakfast also kept convenient breakfast foods on hand in the home (e.g., granola bars, cereal and yogurt) and “*easy on*-*the*-*go snacks*” for eating breakfast in the car. They also taught older children how to prepare simple breakfasts on their own and tried to make eating breakfast a daily expectation or routine (“*get them used to eating breakfast on a daily basis*”). One parent pointed out that “*spousal support* [is] *needed*” to get kids to eat breakfast.

Parents believed that if they ate breakfast and encouraged healthful choices, their preschoolers would be more likely to develop a healthy breakfast routine (“*Parents have to be role models and eat breakfast with the kids*”). Another strategy was to get children involved in decision making (“*Listen to children*’*s opinion as to what they would like to eat for breakfast. Give them options to make them excited about eating*”).

### Sugar-sweetened beverage intake

Parents completing the survey (n = 137) reported drinking one or more sugar sweetened beverages on a mean of 2.7 ± 2.5SD days per week (Table 1). Older parents and those in the highest education group consumed significantly fewer sugary beverages (p = 0.04 and p = 0.02, respectively). Sugar-sweetened beverage consumption did not vary significantly by food security status, geographic location, or primary language spoken.

**Table 1:** **Performance of key weight**-**related behaviors among parents of preschool children**

**Characteristic**	**Percent**	**Days per week parent eats breakfast** **(n** **=** **138)**		**Days per week parent consumes** ≥ **1 sugary drink** **(n** **=** **137)**		**Days per week parent eats** ≥ **2 different fruits** **(n** **=** **138)**		**Days per Week Parent Eats** ≥ **2 Different Vegetables** **(n** **=** **138)**		**Days per week parent plays actively with child (n = 136)**		**Hours per day child watches TV** **(n** **=** **133)**	
		**Mean** **(SD)**	***p***	**Mean** **(SD)**	***p***	**Mean** **(SD)**	***p***	**Mean** **(SD)**	***p***	**Mean** **(SD)**	***p***	**Mean** **(SD)**	***p***
**Total sample**		5.9 (2.2)		2.7 (2.5)		5.2 (1.8)		4.6 (2.0)		4.2 (2.2)		2.4 (1.7)	
**Language**			0.21		0.18		0.08		0.39		0.15		0.07
English	62.6	5.3 (2.7)		2.9 (2.8)		5.4 (1.9)		4.8 (2.1)		4.4 (2.3)		2.2 (1.7)	
Spanish	37.4	5.8(2.1)		2.3 (2.1)		4.9 (1.8)		4.4 (1.7)		3.9 (1.9)		2.8 (1.8)	
Age*			0.44		0.04		0.62		0.49		0.00		0.14
Mean age or younger	44.6	5.7 (2.1)		3.2 (2.5)		5.3 (1.7)		4.5 (2.1)		4.8 (2.1)		2.6 (1.9)	
Older than mean age	54.7	5.4 (2.3)		2.3 (2.5)		5.1 (2.0)		4.8 (2.0)		3.8 (2.1)		2.2 (1.4)	
**Education**			0.63		0.02		0.02		0.40		0.03		0.07
Less than high school	13.7	5.1 (2.7)		2.7^a^ (2.4)		5.0 (2.0)		4.0 (1.8)		5.1^c^ (2.2)		2.5 (1.7)	
High school	20.1	5.2 (2.0)		3.5 (2.5)		5.1 (1.7)		4.5 (1.8)		4.5 (2.3)		3.1 (1.6)	
Some post-secondary education	34.5	5.7 (2.2)		3.1 (2.7)		5.8^b^ (1.4)		4.9 (1.9)		4.6 (2.0)		2.5 (1.9	
Bachelor’s degree or higher	30.2	5.6 (2.2)		1.8^a^ (2.3)		4.6^b^ (2.2)		4.7 (2.3)		3.4^c^ (2.1)		2.0 (1.6)	

*Mean age = 32.18 ± 7.12SD.

Note: For a given characteristic, means in the same column with a common superscript letter are significantly different based on ANOVA post-hoc testing.

### Attitudes toward sugar-sweetened beverage intake

Parents in the 8 focus groups discussing sugar-sweetened beverages (n = 22) felt it was important that their children limit intake of sugary beverages for health reasons (“*obesity rates are increasing*”; “*diabetes is a common problem in most families*”). They recognized that this was important even when kids do not exhibit outward health issues (“*even if kids are skinny*, *it*’*s still not okay*” to drink sugary beverages).

### Perceived barriers to limiting sugar-sweetened beverage intake

Parents indicated key barriers to limiting sugary beverages are availability (“*It*’*s hard to limit because stores have more sugary drinks than healthy drinks*”) and lack of other choices (“*Alternative options to sugary drinks are not as appealing*”). Cost (“*Sugary drinks are very cheap*”, “*It*’*s cheaper to buy soda than juices*”) and financial resources (families “*buy what they can afford*”) also were important barriers. Family priorities (other problems “*might be more important than* … *drinks*”) and desire for a calm home environment (“*Don*’*t want to fight with kids so we just buy them what they ask for*”, “*soda keeps them* [kids] *happy* … *so the kids leave them* [parents] *alone*”) also were named as obstacles to limiting sugary beverages. Some parents felt that a lack of knowledge about these drinks was a roadblock families faced when it came to controlling intake of sweet drinks (“*Many people*…*don*’*t know the amount of sugar and calories in the beverage*”, “*People don*’*t realize how much sugar is in juice*”). Environment (e.g., birthday parties, television advertising) and convenience also presented barriers (“*While on the road*…*a juice box is easier*”).

### Strategies for overcoming barriers to limiting sugar-sweetened beverage intake

To limit sugary drinks, parents reported providing alternative beverages and making them easily accessible to children (“*place water where children can just grab and drink*”). Another tactic was to “*stop buying sugary drinks*”, “*buy less*” and store sugary beverages in inaccessible places (“*Hide the Coke where they can*’*t reach it*”, “*keep soda separate from all other food in the kitchen*”). Other strategies were to control portion sizes of sugar-sweetened beverages (“*only give them a small cup*”), set limits (no seconds allowed, make it a special treat “*once in a while*”), offer other options to control sugar content (“*I give my child artificial sweetener instead of natural real sugar*”, “*water down*” juice, juice drinks, and soda; “*put lemon or lime in water so that the kids think I am giving them lemonade*”, my “*kids think it*’*s* [seltzer] *soda*”), and make healthier beverages more appealing (make “*it fun*, *like smoothies*”, “*put in low cal Crystal Light in their water*”, add “*a scoop of Ovaltine*”).

Still other approaches were to teach their children about “*what is good for them*” (“*let children know* ‘*why*’ *it*’*s important* [to limit sugary drinks]”) and to recognize healthy choices (“*that*’*s not the best choice for your tummy*”). Parents also praised kids for making healthy choices, reasoned with them (“*show them how much it costs to fill cavities*”), and appealed to their sensibilities (“*that food will be better tasting with water*”, sugary drinks will cause you to “*not* [have] *pretty teeth*”, “*to grow taller and grow stronger you need to drink milk*”). Some took a no-nonsense approach to dealing with children’s demands: “*If they are really thirsty and there are no other options for something to drink in the house*, *they can just drink water as a last resort*.”

Parents recognized the importance of role modeling (“*If you don*’*t show your kids how to eat right it*’*s going to be a disaster*”) and outside influences, such as television advertisements, and child care givers (“*if* [day care] *serves sugary beverages* … *parents have no control*”). To overcome problems with access to healthy beverages while away from home, one mother reported that she “*walks around with a cooler full of approved drinks for my kids so that there are healthier options*…*my kids know if they want a snack or a drink*, *they can go there*”.

### Fruit and vegetable intake

On average, surveyed parents (n = 138) reported consuming at least two different fruits and two different vegetables 5.2 ± 1.8SD and 4.6 ± 2.0SD days per week, respectively (Table 1). Number of days per week in which the parent ate at least two different fruits or vegetables did not significantly differ by primary language, geographic location, or age. However, compared to parents with some college, parents with at least a bachelor’s degree (n = 42) were significantly less likely to eat at least 2 different types of fruit each day (p = 0.02).

### Attitudes toward eating fruits and vegetables

Most parents in the 9 focus groups addressing fruits and vegetables (n = 36) felt it was important for children to eat these foods for health reasons (“*play a big part in health*, *growth*, *eyesight*, *and hearing*”, “*help* [the] *digestive system*”). While many knew it was important for health they could not all state what components of health they were beneficial for. Some did not make direct connections to health, but stated fruits and vegetables were “*better* [than] *eating candy*” and “*more important than* … *pizza*.” Others expressed concern about sugar in fruits: “*There are some fruits that are more sugary than others*, *so you have to be careful about what fruits to give them*”.

### Perceived barriers to fruit and vegetable intake

Parents felt that flavor (“*kids don*’*t seem to enjoy the flavor of vegetables*”) and appearance (“*vegetables are not attractive to children*, *they do not look appetizing*”) hindered intake of fruits and vegetables. Varying family preferences (“*my daughter likes it*, *but my son does not*”) also made it difficult to serve these foods.

Cost was cited as another barrier (“*expensive to buy raw vegetables and fruits*”, “*More expensive to have your kids eat healthy all the time* … *crap is cheaper*”) as was lack of planning (“*buys* [fruits and vegetables] *without a plan for the week of what to do with* [them]” so they “*may go to waste*”). Other barriers to fruit and vegetable intake were advertisements for unhealthy foods, no “*commercials for fruits and vegetables*”, and “*characters from TV*/*movies on soda*, *chips*, *cookies*, *et cetera on labels*”.

Finally, portion sizes for fruits and vegetables were difficult for parents. Although most had child-sized dishes and utensils, many were unsure how a preschooler’s portion differs from an adult’s. Parents indicated a desire to learn more about portion sizes.

### Strategies for overcoming barriers to fruit and vegetable intake

To overcome roadblocks associated with taste and appearance, parents served options children prefer, added flavors with condiments “*like lemon*, *ranch*, *and butter*”, sweetened them with sugar, mixed them with other ingredients to hide or improve the flavor of fruits and vegetables (e.g., smoothies, meatloaf, pureed soups), varied cooking methods, and tried cutting fruits and vegetables “*real small*” or into “*fun*” shapes. However, parents indicated the recipes for “sneaking pureed vegetables” in food were not as easy as they had thought, and the recipes took “*lots of work*”,” *too much time*”, and used “*weird ingredients*… *you have to integrate into odd recipes*”.

To overcome the cost barrier, parents used budget planning (“*work with* [your] *budget so you can buy them*”) and sought lower cost purchasing outlets such as farmers’ markets and Asian markets (fruits and vegetables sold in “*some places are cheaper*, *you just have to find them*”). They also “*mapped out a plan*” to use the fruits and vegetables purchased.

Parents offered a variety of learning experiences to help children accept fruits and vegetables, including explaining to kids why eating fruits and vegetables is important (“*I tell my child to eat it to get strong and he believes he will turn out like Superman or a super hero*”), reading books to kids (“*Rah Rah Radishes*”), and letting kids “*play with food so they get to know the texture and flavor*”. Other learning experience strategies were to involve children in food shopping, food preparation (“*If they help you cook*, *they*’*ll eat it*”), planting a garden, and exploring foods together (mother “*shares an apple with him* [son]”).

Parents indicated that the “*important thing is for children to at least try*” fruits and vegetables “*and decide which they like*”. To quell children’s resistance to fruit and vegetable intake, parents gave children choices (provide “*no unhealthy options*”, let them “*freely pick their own*, *instead of forcing them*”) and variety (“*Change up what fruits and vegetables are served so kids don*’*t get bored of them*”). To get children to eat fruits and vegetables, participants thought that parents should “*be persistent*”, be role models (“*If they see you eating it*, *they will eat it*, *too*”), and “*encourage* … *kids to try new healthy foods*.” Some indicated that “*insisting that children eat vegetables is a good idea especially if one offers a reward*” or punishment (“*If you don*’*t eat what*’*s on your plate*, *then no snack*”) whereas others disagreed with this (“*don*’*t push them and make kids angry*; *then they won*’*t eat it and they will be turned off from fruits and vegetables*”). Rewards offered for eating fruits and vegetables included snacks, avoiding punishment, activities (“*family games*…, *renting a movie*, *reading books*”), and praise.

### Healthy portion sizes

Parents in the 11 focus groups (n = 25) exploring portion sizes reported measuring portions or using visual cues to gauge portion size when serving their children. Examples described included using “*very small portions*”, “*cutting adult portions in half*”, giving the younger children in the family “*one less spoonful*” than the older ones, and using “*kid bowls*”, child-sized plates and serving spoons, or measuring cups to serve appropriate portions. Parents described feeding their children by “*trial and error*”, “*estimating*” or “*guessing*”. Parents also noted putting servings of food from each food group on their children’s plates to emphasize variety, letting their children decide when to stop eating, and not forcing their children to eat.

### Attitudes toward serving healthy portion sizes

Parents differed in their attitudes regarding healthy feeding practices. In the focus groups, parents reported it was “important” or “very important” to serve healthy amounts of foods: “*It*’*s very important for kids to learn how to eat the proper amount of food from a young age*”. “*They must eat healthy to be healthy*”. However, others disagreed: “*The amount of food isn*’*t always what is important*…*the quality of the food is the most important factor*”. “*Portions are hard*” and “*difficult to control*”. “*I like my kids to be a little overweight so they can be healthy*”.

Parents were divided about the level of importance to place on serving appropriate portion sizes; some believed portion control was a priority among parents (“*genuine concern*” and “*generally*, [parents] *worry about what their kids eat*”), but others maintained that the opposite was true. “*The people who place more emphasis on nutrition stand out*; *they aren*’*t the norm*”. “*Most parents just guess on portions*”.

### Barriers to serving healthy portion sizes

Parents often cited lack of knowledge of appropriate portion sizes for children (“*it*’*s hard to know the right proportions of each food*”). Many of them stated that they had never really thought about their children’s portion sizes and had never gotten advice about it. Others felt that parental age and upbringing were key. They believed that some cultures, families, and especially “*older parents*” emphasized “*cleaning your plate*” and that parents whose own families had emphasized healthy eating habits would be more likely to do so themselves.

### Strategies for overcoming barriers to serving healthy portion sizes

Parents reported that they would feed their children until they were full, and served children on child-sized plates and cups to keep the amounts they ate under control. Parents also reported monitoring their children’s eating patterns and adjusting subsequent portion sizes accordingly (“*If they only eat 2 chicken nuggets*, *do they ask for a bowl of cereal later*?”). Others compared the amount of food they served to what they saw other parents feeding their children and used it as a gauge to determine whether they were serving appropriate portion sizes.

### Parent feeding practices

Surveyed parents (n = 99), tended to use healthy feeding strategies with their preschoolers. More than one-third (37%) reported using a strategy that encouraged their children to eat by making the food look nice or telling their child that “*food will make you strong*”. This strategy was reported as the main strategy used significantly more often by younger (less than 32 years) and English speaking parents than older and Spanish-speaking parents. Three in 10 parents reported their main feeding strategy was to help children with the process of eating. Another common strategy used by one-eighth of surveyed parents was to let children choose foods he or she wanted to eat from those that have been prepared. Few parents reported physically struggling with their children to get them to eat (1%) or promising their children something if he or she ate (2%) as their primary feeding strategy.

Many parents in the 8 focus groups on feeding practices (n = 17) aimed to avoid arguments over food and instead emphasized keeping mealtimes enjoyable (“*I*’*ll just stop when an argument starts and try again another day*.”). Although on the survey few parents indicated using rewards was their primary feeding strategy, focus group participants reported that they did use rewards for eating, including sweets, toys, activities, television, stickers, and dessert (“*I tell them*, ‘*you will get dessert if you eat what*’*s on your plate*’.”).

### Attitudes toward parent feeding practices

Some parents felt they should require their children to try healthy foods: “*Parents need to be strict and tell child* ‘*you are going to eat this*’.” “*If it*’*s good for him*, *I*’*ll make him try it*.” Other parents felt it was best to encourage, but not force children, to try a new food: “*That*’*s not a good practice*; *you can*’*t force your child to eat*”. “*I don*’*t believe kids need to eat everything or be forced*”.

### Perceived barriers to positive parent feeding practices

Perceived barriers to using positive feeding practices were numerous. Parents cited unhealthy cooking habits (“*cooking with lard*, *oil and butter*”) as common. Some thought that “*broken*” and “*difficult*” homes “*without boundaries*” made feeding children difficult and that busy schedules and the perceived cost and inconvenience of preparing healthy foods caused families to choose less healthy options. Parental frustration with their children’s taste preferences and lack of eating also were given as frequent barriers to trying new or more healthful foods. Parents reported that they “*just want their kids to eat*, *so they give them the food and amounts they want*”, that children “*grow tired of eating things often and become picky*”, or simply “*don*’*t want to eat*”. Concerns about food waste (“*I don*’*t want to throw out food*”) if their children did not like new flavors also were cited. Finally, several parents reported feelings of “information overload” and avoided focusing on specific feeding guidelines. One parent remarked, “*Some people try to follow MyPlate or the Food Guide Pyramid to a* ‘*T*’, *but I don*’*t know. It*’*s hard to translate into real life. You get really overloaded with information. Even though you have all of the information*, *it*’*s what to do with it in the kitchen*”.

### Strategies for overcoming barriers to using positive parent feeding practices

Parents who overcame barriers to positive feeding practices helped their families eat an appropriate amount and avoid overeating by “*feed*[ing] *children when they*’*re hungry at regular mealtimes rather than wait for my husband to get home*”. Parents also suggested having children help with food preparation so that they would “*want to eat what they helped prepare*”. Their tips for dealing with picky eaters included making “*new combos of foods using new and familiar foods*,” introducing new foods multiple times on different occasions, even if they were previously disliked, trying different cooking methods or condiments, adding pureed fruits and vegetables to preferred foods or finding new recipe ideas by searching the internet or speaking with friends. Parents also kept mealtimes and introductions to new foods positive: “*Avoid fights and frustration*” and “*try again after a little while*”. Emphasizing positive characteristics of foods, including flavors, textures, and health benefits, setting a good example by trying foods themselves, and offering praise for healthy behaviors were also mentioned as effective strategies.

### Active playtime behaviors

On average, surveyed parents (n = 136) reported spending 4.2 ± 2.2SD hours per week playing actively with their children. Time spent in active play increased significantly (p = 0.03) when comparing parents who had less than a high school education with those who had a baccalaureate degree or higher and when comparing those younger than 32 years with those who were older (p = 0.00). There were no significant differences in weekly active play based on primary language spoken or geographic location.

### Attitudes toward active playtime

Most parents in the 10 focus groups (n = 28) addressing active playtime felt it was very important for parents to play actively with their children each day (“*should be the number one priority*”). Parents reported improved behavior and sleep habits (“*It*’*s a great way for kids to get all of their energy out so they can be on a steady sleeping schedule*”.), creativity (“*Playing outside*, *they use more imagination*”), and relationships (“*built closer relationships*”, “*being outside helps interactions be more positive*”) when their children had daily active play, particularly outdoors. They also associated active playtime with positive feelings, noting that “*kids enjoy when their parents play with them*”, “*everyone has a memory from the past when they were enjoying themselves outside*”, and “*their faces change*, *and they look so happy*” after playing.

### Perceived barriers to active playtime

Parents consistently cited work, household chores, and lack of time as major barriers to active playtime. Others noted that parents are often “*tired after work*” and “*just want to relax at home*”. Health issues, such as asthma, allergies, and “*physical limitations*” were also common barriers. Space constraints at home (“*live upstairs* [apartment], *so it*’*s hard to have indoor playtime that won*’*t bother the neighbors downstairs*”, “*not enough room to play*”) and concerns that “*it is very easy for things to get broken inside the house*”, or neighbors “*may not like the noise and mayhem* [caused by active play]” were also common. Outdoors, lack of space (“*yard not big enough*”), unsafe surroundings (“*yard does not have a fence or gate*”, “*neighborhood too dangerous for our kids to play outside*”), and poor weather or insects (“*extreme heat*”, “*rain*” and “*mosquitos*”) presented major hurdles. Safe options were often costly; indoor recreation centers (e.g., skating rinks) were expensive, as was the price of transportation (“*too much gas because the park is far*”).

### Strategies for overcoming barriers to active playtime

Parents reported using active gaming (e.g., Wii) indoors and tried a variety of indoor activities (though not all were inherently active), including dance parties, obstacle courses, hide and seek, arts and crafts, and reading books. Some also involved their children in activities they already did, such as walking (“*I walk to the store with my child every night*”), running (“*he sees me run so he runs*”), and yoga. Others suggested community activities (“*soccer and lacrosse*”, “*participate in some kind of sport*”, “[find] *a park within walking distance*”, “[ride] *bikes*”, and “*go to an indoor activities center*”).

### Screen-time behaviors

Surveyed parents (n = 133) reported that their children spent an average of 2.4 ± 1.7 hours per day watching television. Time spent watching television did not differ significantly when comparing parental education, primary language spoken, geographic location or parent age.

### Attitudes toward screen-time

Many of the parents in the 11 focus groups (n = 29) focusing on screen-time behaviors believed it was very important for them to limit screen-time for their children (“*all parents who care will worry*”) but thought other parents might feel limiting television was “*not too important*” or that “*it depended on the person*” (“*every parent has a different perspective on TV time*”) and the age of the child. Parents believed that television helped children “*sit and calm down while the parent takes care of chores*” and was useful as a “*babysitter*” or when the weather was “*very hot to be outdoors*”. Parents also thought television could provide educational benefits if it encouraged active viewer participation (e.g. programs that asked children to “*look for things outside the house*” or “*go to the library*”) and was limited so that “*kids could do things that are more active or socialize with each other*”. In addition, they felt that television viewing needed to be monitored: “*TV is very commercialized*”, “*shows things kids shouldn*’*t watch*” and can lead to “*unacceptable behavior*”, “*obesity*” or “*bad habits*, *like fighting and violence*” because children imitate what they watch. Some, but not all, parents believed that food/beverage advertisements could influence their children (e.g. “*My children think commercials are funny or cool*, *but I will not let it influence them*”). Others thought that television with no or limited commercials or that was geared towards young children was helpful and educational (“[TV watching] *isn*’*t as bad if they*’*re watching educational shows*”).

### Perceived barriers to limiting screen-time

Parents reported that they “*enjoyed the time* [they] *had to get things done when the kids are watching TV*” and found it “*more convenient to do things faster around the house*” such as cooking, cleaning, or other activities that required the parents’ undivided attention. Several cited “*limited availability*” or “*lack of awareness*” of affordable, alternate activities in smaller communities (“*bigger cities offer more opportunitie*s”) and issues with the community (“*parks are not good*”), weather (“*it*’*s too hot during the summer*”), and outdoor safety (“*streets aren*’*t safe*”). Feeling “*too tired*” or “*lazy*” after work, “*working too much to do things with* [their] *kid*”, or needing an easy way to “*get kids out of their hair*” were also common barriers to turning off the television and engaging the family in active playtime.

### Strategies for overcoming barriers to limiting screen-time

To overcome these barriers, parents enrolled children in organized sports, took walks, visited local libraries, museums and zoos and tried “*sharing stories from the day*”. Several parents reported that, by finding “*other avenues of things that tire them*”, their children no longer wanted to watch TV. They also encouraged other parents to seek advice from friends or parent groups and to “*learn new things to play and do with kids*” themselves so that they could be a “*role model*”. They suggested “*not buying a TV*”, “*not watching TV while the kids are awake*”, not placing one in their child’s room, using parental control settings on the television to limit which programs their children watched (e.g. to children’s programming) or setting guidelines for daily screen-time. To avoid the effects of advertisements for unhealthy foods, parents watched and discussed commercials with their children (“*I try to talk about the ads to my child to show them not everything on TV is good for them*.”). Others pre-recorded programs so they could “*fast*-*forward through the commercials*” they did not want their children to view.

### Sleep behaviors

Among surveyed parents (n = 136) in both states, 90% had a set bedtime for their preschoolers. There were no significant differences in whether parents set a bedtime for children with regard to language spoken, parent age, parent education, and geographic location.

### Attitudes toward sleep

In the 9 focus groups (n = 21) examining sleep, all parents agreed that sleep was important and identified improved moods, better performance in preschool and overall growth as benefits (“*The more sleep children get*, *the more productive they are and the more willing to be open*-*minded*”). Negative effects of inadequate sleep included increased irritability (“*moody and tired*”, “*grumpy*”), lack of focus and energy during waking hours (“*sluggish*”, “*don*’*t function well*”), and poor appetite. Some believed that other parents also perceived adequate sleep as essential (“*Never met a parent that doesn*’*t think it*’*s important*”.), but others disagreed (“*Some parents won*’*t set time for them to sleep. They*’*ll just figure the kid will sleep when tired*.”).

### Perceived barriers to sleep

Commonly reported barriers included lack of a bedtime schedule, high emotions before bed (e.g. due to “*school activities in the evening*,” holidays, or family visiting), napping late in the day or for too long, lack of physical activity, eating sweets (“*can hype them up*”), or watching too much television before bed (“*gets so excited that he can*’*t sleep*”). Working parents who came home late reported staying up to spend time with their children (“*If I feel like I haven*’*t spent enough time with my child*, *I*’*ll let them stay up later with me*”). In addition, noise from neighbors or siblings were barriers for some families (“*My seven month old keeps everyone awake at night*, *big time*” or “*Age differences between children make things hard*” and “*My youngest will stay up until 11*, *even though her bedtime is 8 pm*, *because she sleeps in the same room as her sister*”). Other parents reported that their children would only sleep when in the parent’s bed or if the parent was with them (“*I have to get in bed with her to fall asleep*”).

### Strategies for overcoming barriers to sleep

Parents who overcame barriers to sleep found “*flashlights and tons of nightlights*” helped children overcome fears of the dark. Parents also recommended “*scheduling sleep at the same time*” and following a similar routine each evening. Successful bedtime routines varied from having warm baths before bed and playing games during the day (“*soccer*,” “*sports*” and “*walking*”) to tire the preschoolers to turning off the television (“*can*’*t watch Nickelodeon at 11 pm*”) and reading a story or playing soft music, although some parents disagreed and found a silent, dark environment was best (“*no music* – *quiet and* ‘*dungeon*-*like*’” and “*dim the lights*”). Parents also noted that it was important for them to “*be consistent and enforce what they say*” and “*not give in*” when children asked to stay up later.

### Childcare environments

Parents in 10 focus groups (n = 31) identified various factors they considered when choosing childcare for their preschoolers. Trust that staff is well-trained, and a positive, clean environment were most often identified as being important considerations. Other important features when choosing child care situations included strong curriculum, nutritious foods for meals and snacks, opportunities and space for active playtime, scheduled naps, and minimal television viewing.

### Attitudes toward healthy childcare environments

Many parents expected child care to be more structured, like a school (“*Children probably watch television at home*; *children should go to daycare to learn and play*”.). They preferred meals and snacks in childcare settings that were “*healthy*” and “*nutritious*” with a variety of foods and options for parents to pack lunches for their children. The quality of the education and instructors and the inclusion of nutrition lessons was important, as well (“*What do they do with the child during the day*? *I want the child tired when they come home*”. “*Teaching nutrition at their level is important*”.). Some perceived that other parents also prioritized “*healthy foods*”, a variety of educational and active playtime activities and “*little or no TV watching*” when considering childcare options. However, some disagreed and believed that, for many other parents, location, cost and convenience were probably more important than the curriculum (“…*a lot of parents don*’*t care*; *they look at daycare as babysitting*”.).

### Perceived barriers to healthy environments in childcare

Cost and convenience were frequently given as barriers to choosing centers with more healthful options (“*Some parents need free care*, *so they really don*’*t have much of a choice.*”). Lack of interest in healthy foods among childcare staff was another barrier (“*I did go and ask the owner to give the kids more veggies and foods without grease*, *but they never did change the menu*”). Parents also felt that some childcare centers focused more on recruiting and enrolling students than on “*meeting the needs of the parents and children*” by providing healthy options.

### Strategies for overcoming barriers to childcare

Parents who overcame barriers to healthy environments at childcare centers used comment cards or tried to work with staff to improve the facility’s environment for their preschooler (“*Communication is important*.” “*Ask the reason why they are doing that*.” “*Discuss*…*other options to help the daycare make changes*.”). Parents also reported sending lunches and snacks from home so they could control what their children ate when in childcare.

## Discussion

Although many preschool parents understand the importance of eating healthfully and engaging in positive, weight-related behaviors, they also encounter a variety of barriers to making these practices a routine and could benefit from additional information that is applicable to families with children the same age. Similar challenges have been reported in other studies among parents of both school-aged children [65,66] and preschoolers [67]. In particular, lack of time for healthy cooking and eating together [65,66,68,69], confusion about correct serving sizes for preschoolers [70], and difficulty finding safe places or activities for active play [71] are typical barriers that were expressed by parents in the present study, as well. However, parents also are eager to share strategies that work [72].

Given the variation in lifestyles and barriers experienced by each family, parents of preschool children should be engaged to help educators develop relevant messages encouraging families to adopt healthy routines tailored to their household’s needs [67]. For example, participants in the present study indicated time was a major barrier to breakfast consumption and that time management strategies were helpful. Although there are limited data about breakfast consumption among preschoolers and their parents, research with older children (4^th^-6^th^ graders) has also identified lack of time as a barrier to breakfast [73]. In addition, when eating dinner as a family is not a viable option, family breakfasts also have positive associations with weight and nutrient intake [74].

Previous research has reported that parents choose sugary drinks based on cost, taste preferences, and a desire to please their children and maintain a calm home environment [75] and that socioeconomic factors such as education and income [76,77] may affect intake of sugary drinks. A study of more than 2000 Canadian preschoolers found that those in low- and medium-socioeconomic status neighborhoods were significantly more likely to drink regular soft drinks when compared with children from neighborhoods with a higher socioeconomic status [77]. In the present study, parents with more education were less likely to consume sugary drinks; however they also were less likely to eat two different types of fruit each day. Others have reported either a positive or no relationship between parental education and fruit consumption among parents and older children [78,79]. Given the mealtime challenges reported by preschool parents, barriers such as lack of time for food preparation, eating “on the run”, or issues with picky eaters may make eating a variety of fruits each day and consuming fewer sugary drinks challenging. Data from the current study indicating parent need for convenient beverages, uncertainty about how to prepare fruits and vegetables in appealing ways, questions about 100% fruit juice, and confusion about portion size all suggest areas to focus on in future interventions.

Positive parent feeding practices are an important way to tie together healthy diets in a way that is most beneficial to preschool children’s learning how to feed themselves and make healthy choices as they age. In the current study, parents described using a variety of feeding practices, including instrumental feeding (e.g. offering dessert as a reward for eating the main course), pressure to eat, and less rigid prompting to eat [80]. Education focused on teaching parents appropriate portion sizes, cooking methods, tips for cooking with preschoolers and ideas for food combinations to help them introduce new foods may address some of the barriers parents described with regard to feeding practices. Although few interventions have focused on improving feeding practices, [81,82] education efforts that provide an understanding of healthful feeding practices could engage families to share successful strategies [83,84].

In addition, parents in the current focus groups reported finding safe spaces for active playtime was a major challenge. Concerns about strangers, road safety, area deprivation, and crime have been previously identified as possible barriers to children’s physical activity [71,85], although empirical data on how perceived safety affects active playtime are mixed [85,86]. Surprisingly, although parents in the current study mentioned high temperatures as a barrier to outdoor play (especially among parents in Arizona), a previous report found preschoolers spent the least time outdoors during months with pleasant temperatures and that preschool boys spent more time outdoors during hotter months [87]. Thus, factors beyond weather and perceptions of safety likely influence active play.

In the present study, younger parents and those with less education reported spending more time in active play with their children compared to those older than 32 or with higher educational attainment; although there is limited research with preschool parents in this area, one study found a positive trend between parent education and preschooler physical activity and a negative trend with parent physical activity [88,89]. Although older parents and those with more education may have more resources to purchase sports equipment or pay for participation in athletic programs, their work schedule may reduce leisure time with their families. Education programs may thus need to emphasize simple activities that can be performed indoors or outdoors, depending on weather and the outdoor environment. Programs should be tailored to meet the needs of parents in a particular neighborhood because barriers and supports appear to vary, based on a variety of factors, that may be specific to a particular community.

In contrast to limited physical activity participation, television viewing among preschoolers of focus group parents was greater than the “<1 to 2 hours per day” of screen-time recommended by the American Academy of Pediatrics [90]. Not surprisingly, many of the supports for increased screen-time, including limited alternative activities, concerns about weather and safety issues related to playing outdoors, fatigue after work, and the need for an easy “babysitter” while parents completed chores, were mentioned as barriers to physical activity. This is supported by a report finding that preschoolers who lived in neighborhoods perceived by their mothers as least safe were more likely to watch more than 2 hours of television each day [86]. In the present study, focus group parents generally believed it was important to limit screen-time, but the survey data indicate most did not actually do so. Given the connection between barriers to active playtime and limiting screen-time, interventions should target both ways to reduce screen-time and increase active playtime while emphasizing the guidelines for reduced television viewing. In particular, ideas for activities that children can do independently or get children involved in household chores (e.g. food preparation) may benefit parents who turn to the television for babysitting.

Knowledge and use of practices recommended for optimal sleep hygiene [91] were common among focus group parents. However, data suggest that sleep problems are common in preschoolers [92]. Parents may benefit from tips and strategies that help them establish bedtime routines with their preschoolers as well as educational opportunities that help them learn the importance of a regular bedtime for good health.

Finally, participants’ desired qualities in a childcare center mirrored many of the concerns expressed in other areas (e.g. the importance of prioritizing healthy foods, encouraging active play, and limiting screen-time). However, many parents were compelled to choose centers they believed were less-than-ideal due to cost or convenience and found the staff unsupportive or not interested in promoting healthy behaviors. Although attending childcare has been shown to protect against obesity [26], a review of nutrition practices in childcare centers reported that programs such as Head Start were likely to serve more healthful foods and encourage healthy behaviors whereas smaller and family-run centers were not [31]. Research on factors that strengthen relationships between parents and staff in early childhood programs has identified communication as an important feature of high quality programs that can help staff and families establish and work towards shared goals [93]. It is therefore important that parents feel empowered to communicate their wishes and advocate for healthier child care when feasible. In addition, future research could examine whether the childcare center qualities parents desire vary by type of center (e.g. Head Start versus home-based programs), socio-economic status and days per week in childcare.

It is interesting to note that while health is an important reason why parents believe key weight-related behaviors to be important, other inputs appear to hold as much if not more importance. Time, money, family happiness, family cohesion, and calm home environments were often mentioned as reasons why parents reported both healthy and unhealthy behaviors (“…*giving them soda keeps the kids happy* … [parents] *might* [give kids soda] *so the kids leave them alone*”, “…*money issues might be more important*…”), which is consistent with other findings [83]. Given that participants were recruited through advertisements indicating the study’s purpose was to help children grow up healthier, it is possible that they may have been more health-conscious than parents in the general population.

Although the findings of this study are limited by the sample size, it is one of the first studies to qualitatively examine cognitions of parents with regard to a broad array key weight-related behaviors. The findings can inform the future development and evaluation of interventions aiming to help parents raise children with healthy body weights. Future work should explore these findings in other states, with a larger and more diverse sample.

## Conclusions

There is substantial evidence that the key weight-related behaviors investigated here are important mediators of obesity risk for preschool children and their parents [22,94-97]. Parents are a key modifier of the environment of young children and also serve as role models for behavior development [9,13-21] The Social Cognitive Theory emphasizes the importance of environment and behaviors working in tandem to promote healthy changes [98,99]. Increasing self-efficacy to make such changes is also an important construct of the Social Cognitive Theory [100]. This study reinforces that social modeling, or “showing the person that others like themselves can do it,” can be an important technique to increase self-efficacy [101], p. 177. As adult learners, parents bring their own life experiences and knowledge upon which they base their behaviors and modify environments [102]. Although from different backgrounds and different life experiences, adopting the attitudes and techniques for overcoming barriers that have been used by peers (e.g., other parents of preschoolers), can be an important method to promoting weight-related healthy behavior change in parents and their preschool children and could be an important addition to obesity-prevention programs that help parents “*translate it into real life*”.
